# LONGITUDINAL ASSOCIATIONS BETWEEN LONELINESS AND DEPRESSIVE SYMPTOMATOLOGY IN THE OLDEST-OLD

**DOI:** 10.1093/geroni/igae098.3503

**Published:** 2024-12-31

**Authors:** Morgan Lynch, Christopher Beam

**Affiliations:** University of Southern California, Los Angeles, California, United States; University of Southern California, Los Angeles, California, United States

## Abstract

Prior research suggests that loneliness is a precursor for depressive symptomatology (hereafter “depression”) in older adults (Cacioppo et al. 2010). Yet, whether their findings extend to the oldest-old is an open question. Moreover, recent work suggests that loneliness does not predict depression after accounting for trait-level components of each variable (Griffin et al., 2022). In the current study, we model five waves of loneliness and depression scores, measured two years apart, from 702 pairs of Swedish twins 80+ years of age from the Origin of Variances in the Oldest-Old study (OCTO). We use a Trait-State-Error (TSE) model to estimate trait, correlated time-specific, and uncorrelated time-specific components of loneliness and depression in the oldest-old. Lag-1 phenotypic correlations between loneliness and depression were small to moderate (rs:.23-.42). Univariate models were first compared; a State Error (SE) model best fit both variables. Model comparison tests indicated between-person differences (i.e., trait-like components) were not significant for either loneliness or depression. In a bivariate SE model, we found bidirectional cross-trait effects; depression positively predicted loneliness (b = 0.19, 95% CI [0.03, 0.46]), and loneliness negatively predicted depression after accounting for within-timepoint correlations (b = -0.33, 95% CI [-0.61, -0.09]). Within-variable autoregressive components were also significant; higher scores at the previous timepoint predicted an increase in loneliness (b = 0.91, 95% CI [0.84, 0.96]) and depression (b = 1.19, 95% CI [1.11, 1.27]). Findings suggest that in the oldest-old, loneliness does not presage depression as it does in young-old, alternatively, depression may precede loneliness.

